# A Protoplast Transient Expression System to Enable Molecular, Cellular, and Functional Studies in *Phalaenopsis* orchids

**DOI:** 10.3389/fpls.2018.00843

**Published:** 2018-06-22

**Authors:** Hsiang-Yin Lin, Jhun-Chen Chen, Su-Chiung Fang

**Affiliations:** ^1^Biotechnology Center in Southern Taiwan, Academia Sinica, Tainan, Taiwan; ^2^Agricultural Biotechnology Research Center, Academia Sinica, Taipei, Taiwan

**Keywords:** protoplast, *Phalaenopsis aphrodite*, orchid, transient expression, gene regulation

## Abstract

The enigmatic nature of the specialized developmental programs of orchids has fascinated plant biologists for centuries. The recent releases of orchid genomes indicate that orchids possess new gene families and family expansions and contractions to regulate a diverse suite of developmental processes. However, the extremely long orchid life cycle and lack of molecular toolkit have hampered the advancement of orchid biology research. To overcome the technical difficulties and establish a platform for rapid gene regulation studies, in this study, we developed an efficient protoplast isolation and transient expression system for *Phalaenopsis aphrodite*. This protocol was successfully applied to protein subcellular localization and protein–protein interaction studies. Moreover, it was confirmed to be useful in delineating the PaE2F/PaDP*-*dependent cell cycle pathway and studying auxin response. In summary, the established orchid protoplast transient expression system provides a means to functionally characterize orchid genes at the molecular level allowing assessment of transcriptome responses to transgene expression and widening the scope of molecular studies in orchids.

## Introduction

Orchidaceae represent one of the largest angiosperm families comprising more than 25,000 species that are grown in a wide range of habitats including rainforest, grassland, and even mangrove swamp and low arctic tundra. Orchids have distinct morphological and physiological characteristics such as the co-evolution of pollinators and distinct floral structure (Waterman and Bidartondo, 2008), lack of cotyledon development during embryogenesis (Kull and Arditti, 2002), formation of pollen dispersal units (pollinia) (Pacini and Hesse, 2002), and unique growth and development coupled with mycotrophic strategies (Rasmussen, 2002). These unique developmental programs or strategies have drawn the attention of many evolutionary and plant biologists. Additionally, the wide use of certain orchids as medicinal plants indicates that orchids may have a repertoire of secondary metabolites whose functionality still remains to be explored. (Kong et al., 2003; Bulpitt et al., 2007; Bory et al., 2008). Despite the enormous interest in understanding the molecular mechanisms of the specialized developmental or physiological programs in orchids, the lack of a robust molecular toolkit hampers the advancement of orchid biology.

Recent efforts using next generation sequencing have started to unravel the complexity of the orchid genome and transcriptome atlas (Rao et al., 2014; Cai et al., 2015; Fang et al., 2016; Zhang et al., 2016, 2017; Chao et al., 2017). Progress in the development of tools for manipulation and analysis of cellular processes has promoted research in various orchid species (Yu et al., 2001; Liau et al., 2003; Hsu et al., 2011; Lu et al., 2012; Chen and Fang, 2016; Chen et al., 2016; Hsing et al., 2016). However, studies of orchid gene functions and genetic networks are highly challenging because of a lack of mutant collections and the large amounts of time needed to obtain transgenic orchids.

Protoplast transient expression systems have been widely used to study gene regulation, protein localization, protein–protein interactions, and cell signaling pathways in response to hormones, environmental cues, and pathogen-derived elicitors in model systems (Sheen, 2001; Fraiture et al., 2014). Because of their versatility and ability to detect cell-autonomous responses, protoplast transient expression systems have also been developed and applied to many non-model plants whose transformation platforms are not yet available or for which regeneration of transgenic plants is difficult (Nyman and Wallin, 1992; Hirata et al., 2012; Bu et al., 2014; Lin Y.C. et al., 2014; Kidokoro et al., 2015; Muchero et al., 2015; Kanofsky et al., 2016; Lu et al., 2016; Nanjareddy et al., 2016; Thevenin et al., 2016; Shen et al., 2017; Wang et al., 2017). In addition, protoplast-based transient expression systems allow the study of immediate transcriptome responses to expression of the genes of interest and provide an alternative means to characterize and analyze the cellular functions and regulatory networks of such genes.

The protoplast system has also been used to investigate and discover signaling transduction pathways in various plants (Sheen, 2001; Asai et al., 2002; Baena-Gonzalez et al., 2007; Müller and Sheen, 2008; Boudsocq et al., 2010; Fraiture et al., 2014). Even though protoplast isolation has been reported in *Phalaenopsis* orchids, the requirement of callus induction for protoplast preparation makes the procedure difficult to implement (Kobayashi et al., 1993; Shrestha et al., 2007). A recent study reported a protoplast-based transient gene expression protocol in a *Phalaenopsis* hybrid cultivar (Li et al., 2018). Inconveniently, this protocol requires young leaves of shoots induced from flower nodal buds that may not be readily available in different *Phalaenopsis* cultivars. Also, the transfection efficiency is below 50%, a minimum threshold (Yoo et al., 2007) required to obtain reliable and repeatable data for molecular studies. Furthermore, the protoplast transient expression protocol and its broad usage for functional genomics studies have not been rigorously tested. To simplify the protoplast preparation procedure and establish a system for rapid gene regulation studies for orchids, here an optimized petal-based protoplast isolation and transient expression protocol was established. This protoplast transient expression system worked successfully in investigating subcellular localization of proteins and protein–protein interaction. In addition, our results demonstrate its amenability for studies of transcription activity of PaE2F3/PaDP transcription factors and auxin response. Taken together, development of an orchid protoplast transient expression assay provides a versatile experimental platform to enable molecular, cellular, and functional studies of orchids. Because experimental settings and empirical experience are provided, the testing parameters may easily be adjusted for different orchid species.

## Materials and Methods

### Plant Materials and Growth Conditions

Tetraploid *Phalaenopsis aphrodite* subsp. *formosana* (m1663) plants in 3.5-inch pots were purchased from Chain Port Orchid Nursery (Ping Tung, Taiwan). Plants were grown and maintained as previously described (Chen and Fang, 2016). Under flowering inductive conditions [alternating 12 h light (23°C)/12 h dark (18°C) cycles], the floral stalks (∼0.5 to 1 cm long) became visible approximately 2 months after treatment. The first open flower appeared approximately 3–4 months after treatment.

### Protoplast Isolation

Fully open flower petals were used for protoplast isolation. Petal protoplasts were successfully isolated from petals collected 1–15 days after full bloom. Orchid petals were cut into 0.5–1.0-mm strips using a fresh sharp razor blade. The petal strips were transferred to a petri dish containing freshly prepared enzyme solution. The enzyme solution was made as follows: 1% (w/v) cellulase R-10 (Yakult Pharmaceutical), 0.25% (w/v) macerozyme R-10 (Yakult Pharmaceutical), 0.7 M (or otherwise described in the Results) mannitol (Sigma), 20 mM KCl (Sigma), and 20 mM MES (pH 5.7, Sigma) were warmed up to 55°C for 10 min to enhance enzyme solubility and to inactivate DNase and protease. The enzyme solution was allowed to cool to room temperature before 10 mM CaCl_2_ and 0.1% BSA (Sigma cat #A7906) were added. The enzyme mixture was then filtered and sterilized by 0.45 μm Millex-HP filter (Millipore). The petal strips were then completely submerged in the enzyme mixture and allowed to digest without agitation in the dark for approximately 16 h (or as described in the section “Results”). Carbenicillin was added to a final concentration of 50 μg/ml to avoid bacterial contamination. Adding carbenicillin during the protoplast preparation is strongly recommended for petals collected from the greenhouse.

After digestion, the enzyme mixture was gently agitated to release the protoplasts and the protoplast/enzyme suspension was diluted with equal volume of wash and incubation solution (WI-0.7) that contained 0.7 M mannitol (or as described in the section “Results”), 20 mM KCl, and 4 mM MES (pH 5.7). The protoplast/enzyme solution was then filtered through a 100-μm nylon mesh (BD Falcon) to remove tissue debris. (Note: the mesh is normally kept in 95% ethanol and rinsed with WI-0.7 solution before use). The flow-through was then centrifuged at 200 *g* for 2 min in a desktop centrifuge (Eppendorf 5810R) to pellet the protoplasts. The acceleration ramp was set to 2 and deceleration ramp was set to 0. The supernatant was removed and the pellet was gently resuspended in 3 ml WI-0.7 solution. The protoplast suspension was washed gently one more time with 3 ml WI-0.7 solution. The cell concentration was measured using a hemocytometer. The protoplast suspension was kept on ice for 30 min. The protoplast suspension was briefly centrifuged at 200 *g* to pellet protoplasts. WI-0.7 solution was carefully removed and the pellet was resuspended in pre-chilled MMG-0.7 solution (0.7 M mannitol, 15 mM MgCl_2_, and 4 mM MES, pH5.7) to obtain a cell concentration of approximately 1.0 × 10^6^/ml. Based on our protocol, 20 ml of enzyme solution can digest up to 20 orchid petals (10 flowers) and yield 5 ml of ∼1.0 × 10^6^/ml protoplasts before transfection. Approximately 1.0 × 10^6^ cells (from 4 petals) are required for each RNA preparation.

### DNA-PEG-Calcium Transfection

A modified PEG-mediated protoplast transfection protocol (Yoo et al., 2007) was used. Orchid protoplasts were adjusted to a final concentration of ∼0.5–2 × 10^5^ cells/ml with MMG-0.7 solution. Twenty microliters of 10 to 20 μg plasmid DNA was mixed with 200 μl protoplasts (∼1–4 × 10^4^ cells) in MMG-0.7 solution and an equal volume (220 μl) of freshly prepared PEG-calcium transfection solution was added. PEG-calcium transfection solution (40% w/v PEG4000, 0.6 M mannitol, and 0.1 M CaCl_2_) was prepared as follows: PEG4000 (Fluka, cat. no. 81240) and mannitol were first dissolved in water by heating up to 60°C for approximately 10–20 min. After the solution was cooled down to room temperature, CaCl_2_ was added. The DNA-PEG-calcium-protoplast solution was mixed gently and incubated at room temperature for 6–10 min. After transfection, the transfected protoplast mixture was immediately diluted with 2–3 ml of WI-0.7 solution and centrifuged at 200 *g* for 2 min. Then the supernatant was carefully removed. The transfected protoplasts were washed one more time with 2–3 ml of WI-0.7 solution followed by centrifugation. The protoplasts were gently resuspended in 1 ml WI-0.7 solution. The protoplast mixture was carefully removed and the transfected protoplasts were incubated in WI-0.7 solution in 12-well tissue culture plates or Eppendorf tubes pre-rinsed with 1% BSA solution for the desired amount of time before further analysis. It is recommended that ∼1 × 10^6^ cells/ml are used and the amount of plasmid DNA is scaled up if transfected protoplasts need to be harvested for RNA or protein extraction.

For hormone treatment, transfected protoplast cells were treated with 1 μM 1-naphthaleneacetic acid (NAA), 100 nM *trans*-zeatin, 50 μM gibberellic acid 3 (GA_3_), or 100 μM abscisic acid (ABA) (Müller and Sheen, 2008) for 2 h before microscopic observation. Fluorescence images were photographed on a LSM 710 Confocal Microscope (Zeiss) or Zeiss Axio Scope A1 microscope equipped with an AxioCam HRc camera.

### Protoplast Viability Test

Propidium iodide (PI) was dissolved in 0.65 M mannitol to make 0.5 mg/ml stock solution. Fluorescein diacetate (FDA) was dissolved in acetone to make 5 mg /ml stock solution. A total of 20 μl of PI and 20 μl FDA stock solutions were added in 1 ml 0.65 M mannitol as the staining solution (this has to be made fresh). For staining, 10 μl of staining solution was added into 20 μl of isolated protoplast cells and incubated at room temperature for 1–2 min. The living protoplast cells (green, stained with FDA) and dead cells (red, stained with PI) were visualized and photographed by LSM 710 confocal microscope (Zeiss). Five to twelve snapshots were taken for each sample. For viability measurement, at least 150 protoplast cells were examined from each sample. Viability was measured as green cells/green + red cells × 100%. These experiments were repeated at least three times.

### RNA Isolation and Quantitative RT-PCR

The transfected protoplasts were flash frozen in liquid nitrogen and stored in a freezer at –80°C. RNA was isolated using RNA extraction reagent (3-Zol, MDBio, Inc.) according to the manufacturer’s instructions. To remove DNA, total RNA was treated with RNase-free DNase (Qiagen) followed by RNeasy column purification (Qiagen) according to the manufacturer’s instruction.

RNA (0.4 to 1 micrograms) was reverse transcribed in the presence of a mixture of oligo dT and random primers (9:1 ratio) using the GoScript Reverse Transcription System (Promega) as described previously (Lin H.Y. et al., 2014). Ten microliters of quantitative RT-PCR reaction contained 2.5 μl of 1/20 diluted cDNA, 0.2 μM of primers, and 5 μl of 2X KAPA SYBR FAST master mix (KAPA Biosystems). Real-time RT-PCR was carried out using a Bio-Rad CFX96 (Bio-Rad). The following program was used for amplification: 95°C for 1 min, 40 cycles of 95°C for 5 s, and 58°C for 20 s. PCR was performed in triplicate, and the experiments were repeated with RNA isolated from three independent samples. Fold change in expression was calculated as 2^-ΔΔCT^. A melting curve of each PCR was examined to ensure no spurious products were present. Primer pairs used for quantitative PCR are listed in Supplementary Table S1. Because expression level of ubiquitin (*PaUBI1*) remained relatively constant across the tissues examined (Lin H.Y. et al., 2014), it was used as an internal control.

## Results

### Protoplast Isolation

**FIGURE 1:**
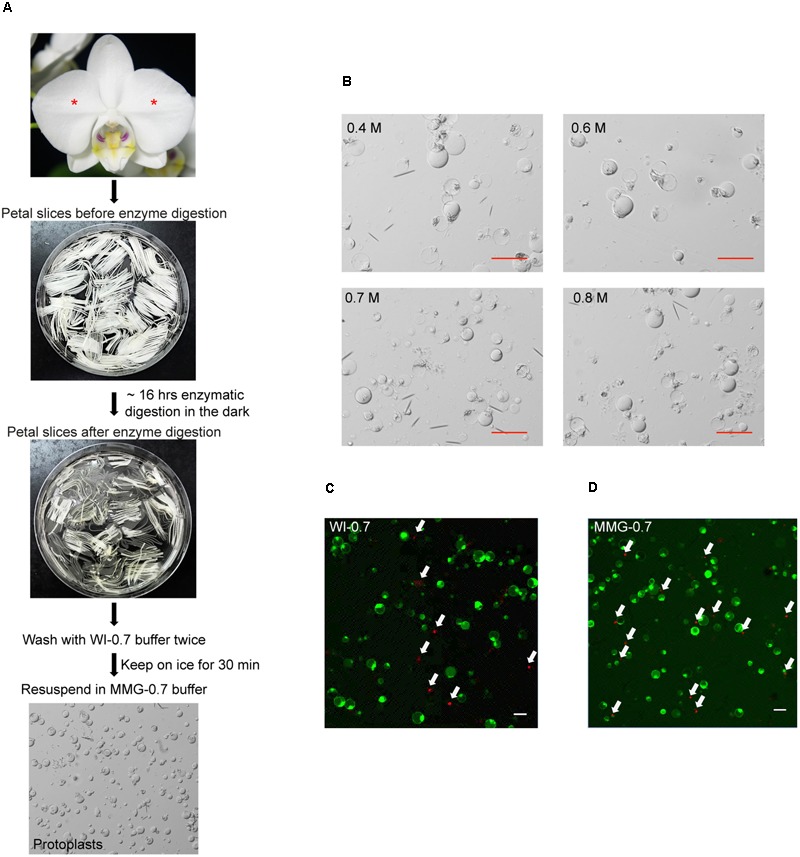
Isolation of protoplasts from petals of *P. aphrodite*. **(A)** A flowchart showing the procedure of protoplast isolation. Asterisks mark the petals used for protoplast isolation. **(B)** Protoplast morphology remained intact in enzyme solution supplemented with 0.4 M, 0.6 M, 0.7 M, or 0.8 M mannitol. **(C)** A snapshot of cell viability test after resuspending cells in WI-0.7 solution. **(D)** A snapshot of cell viability test after resuspending cells in MGG-0.7 solution. White arrows indicate the dead cells stained by PI. Red and white scale bars, 50 μm.

Petal protoplast isolation has been reported in *Dendrobium* orchid (Hu et al., 1998). We therefore chose petals of *Phalaenopsis aphrodite* as our starting materials. The release and integrity of petal protoplasts was visually inspected in cellulose- and macerozyme-containing enzyme solution adjusted to different osmotic conditions (in 0.4 M, 0.6 M, 0.7 M, or 0.8 M mannitol). Protoplasts were successfully released from petal tissues and remained intact after overnight (∼16 h) enzyme digestion (**Figure 1A**) regardless of the concentrations of mannitol tested (**Figure 1B**). To survey the viability of the petal protoplasts, PI and FDA, which mark dead and live cells, respectively (Huang et al., 1986), were used to stain the isolated protoplasts. Petal tissue digested with enzymes for 16 h gave a better yield than that digested for only 8 h (**Table 1**). Moreover, the protoplast viability was not compromised after 16 h of digestion (**Table 1**). Approximately 90–94% of protoplasts were viable after resuspension in WI buffer supplemented with various concentrations of mannitol (**Table 2** and **Figure 1C**). More than 80% of protoplasts remained viable after resuspension in MMG-0.6 and MMG-0.7 solution (**Table 2** and **Figure 1D**). Approximately 75.3% and 77.5% of protoplast cells prepared in 0.6 M and 0.7 M mannitol-based solution, respectively, remained viable after the transfection procedure (**Table 2**). Even though there was no drastic difference in cell viability with preparation in 0.6 M or 0.7 M mannitol-based buffer, 0.7 M mannitol seemed to work slightly better in protecting cells during the transient expression procedure. Therefore, 0.7 M mannitol was chosen for the rest of the experiments. Under this condition, the yield of viable protoplasts from one petal after transfection was estimated to be approximately 1.9 × 10^5^ cells [2.5 × 10^5^ cells (number of protoplast cells per petal) × 77.5% (viability of transfected protoplast prepared in 0.7 M mannitol condition)]. The size of petal protoplasts was calculated and it ranged from 20 to 50 μm in diameter with an average of approximately 34 ± 7.1 μm in diameter (Supplementary Datasheet S1). These results demonstrated the feasibility of using orchid protoplasts for further molecular biology analyses.

**Table 1:** Effect of duration of enzyme digestion on protoplast yield and viability.

Digestion duration in enzyme solution supplemented with 0.7 M mannitol	Protoplast Yield (×10^5^ cells/ petal)	Viability (%)
8 h	2.2 ± 0.4	89.1 ± 3.3
16 h	6.1 ± 0.5	90.1 ± 2.4

*^∗^Standard deviations were derived from three independent experiments.*

**Table 2:** Effect of mannitol concentration on protoplast viability during the isolation procedure.

After enzyme digestion and resuspension in WI based buffer	Viability (%)
0.4 M	90.7 ± 5.7
0.6 M	91.6 ± 4.5
0.7 M	91.1 ± 6.1
0.8 M	94.1 ± 2.1

**After washing and resuspension in MMG based buffer**	**Viability (%)**

0.4 M	77.3 ± 10.5
0.6 M	81.1 ± 8.2
0.7 M	81.3 ± 0.4
0.8 M	73.9 ± 9.2

**After transfection**	**Viability (%)**

0.4 M	66.6 ± 2.6
0.6 M	75.3 ± 4.2
0.7 M	77.5 ± 2.4
0.8 M	67.9 ± 12.4

*^∗^Standard deviations were derived from at least three independent experiments.*

Leaves are readily accessible and mesophyll protoplast cells have been successfully isolated from leaf tissues of various plant species (Sheen, 2001; Chen et al., 2006; Yoo et al., 2007; Mazarei et al., 2008; Lung et al., 2011; Masani et al., 2014; Nanjareddy et al., 2016; Shen et al., 2017). Therefore, we also tested the conditions for mesophyll protoplast preparation. The youngest fully expanded leaves were used for this test. Similarly, different osmotic conditions of enzyme solution (in 0.4 M, 0.6 M, 0.7 M, or 0.8 M mannitol) were tested and the integrity of leaf mesophyll protoplasts during enzyme digestion was inspected under a microscope over time. Unlike petal protoplasts, the integrity of mesophyll protoplasts declined quickly over time. For the cells that retained relative integrity, the interior content of protoplasts including chloroplasts was gradually concentrated and pushed to one side of the cell (Supplementary Figure S1A). Mesophyll protoplast cells started to rupture after 2 h of incubation in enzyme solution (Supplementary Figure S1B). The cell integrity was completely disrupted after transfection (Supplementary Figure S1C).

### Protoplast Transient Expression System Enables Subcellular Localization and Bimolecular Fluorescence Complementation Studies

**FIGURE 2:**
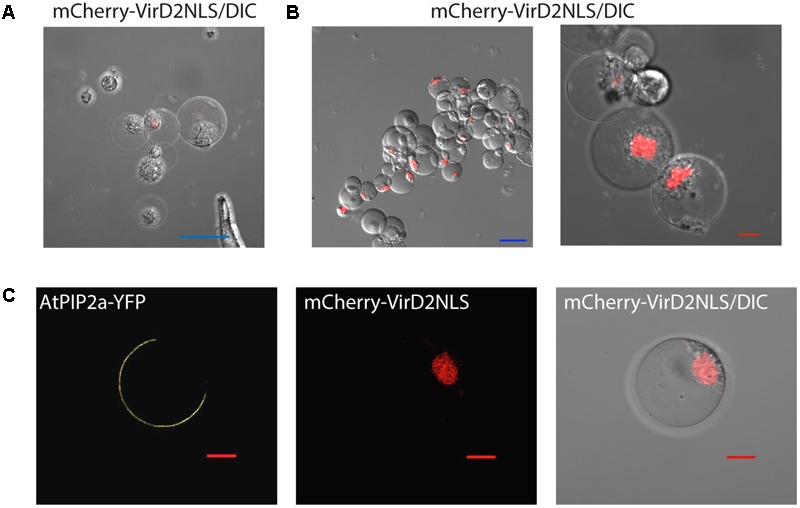
Subcellular localization of protein markers. **(A)** Nuclear localization of mCherry-VirD2NLS marker 4 h after transfection. **(B)** The vast majority of cells expressed the nuclear marker 20 h after transfection. **(C)** Plasma membrane localization of AtPIP2a-YFP aquaporin marker. Note that the protoplasts were co-transfected with AtPIP2a-YFP and mCherry-VirD2NLS markers. Blue scale bar, 50 μm; red scale bar, 10 μm. DIC, differential interference contrast image of cells superimposed with the fluorescence marker.

To test whether petal protoplasts were suitable for subcellular localization studies, nuclear and plasma membrane markers were transformed into protoplasts by PEG-mediated transformation (see Methods). As expected, nuclear marker mCherry-VirD2NLS (Lee et al., 2008), which carries the nuclear localization signal, started to appear in the nucleus 4 h after transfection (**Figure 2A**) and accumulated in almost all the inspected nuclei 20 h after transfection (**Figure 2B**). Unlike biolistic transient assay where cells with fluorescence signals were sparsely distributed on the petal due to uneven spraying of the gold particles (Supplementary Figure S2), the majority of protoplasts (>80%, Supplementary Datasheet S2) had fluorescence signals, indicating protoplast-based transfection is relatively homogenous and enables broader molecular and biochemical analyses of the transgene-encoded protein product (**Figure 2B**). This transfection efficiency is almost equivalent or better than protoplast transfection efficiency reported in the model systems (Yoo et al., 2007; Wu et al., 2009; Faraco et al., 2011). In addition, the plasma membrane marker aquaporin AtPIP2a-YFP (Nelson et al., 2007) was correctly targeted to the plasma membrane (**Figure 2C**). These experiments demonstrated that *Phalaenopsis* orchid protoplasts are suitable for protein localization studies.

**FIGURE 3:**
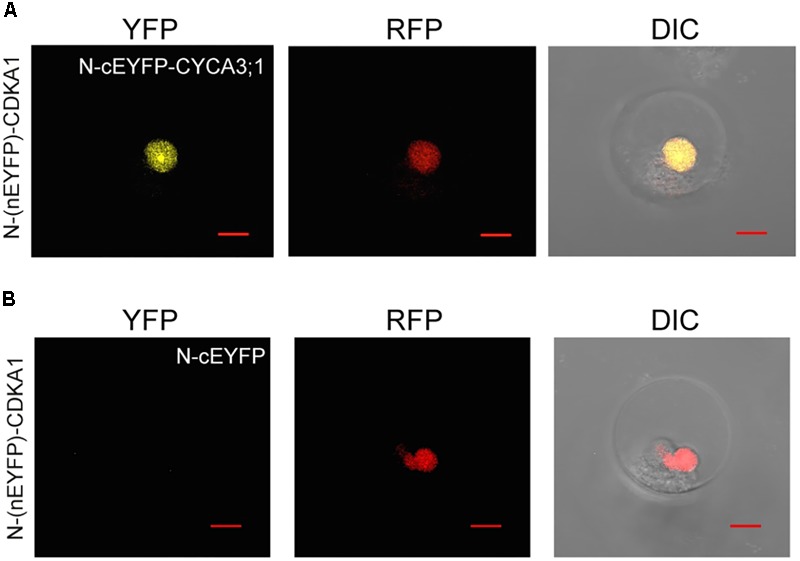
Protein–protein interactions of CDKA1/CYCA3;1 proteins visualized using BiFC. **(A)** The BiFC signal was detected in protoplast cells co-transfected with *N*-*(nEYFP)*-*CDKA1* and *N*-*cEYFP*-*CYCA3;1* constructs. **(B)** No signal was detected in protoplast cells co-transfected with *N*-*(nEYFP)*-*CDKA1* and *N*-*cEYFP* constructs. mCherry-VirD2NLS was used as a nuclear marker. DIC, differential interference contrast images of cells superimposed with YFP and RFP channels. Scale bar, 10 μm.

The application of petal protoplasts to bimolecular fluorescence complementation (BIFC) analysis for protein–protein interaction study was also tested. Cyclin-dependent kinases (CDKs) are central cell-cycle regulators whose activities are regulated by physical interaction with the cell-cycle phase specific cyclins (CYCs) (Morgan, 2007). To test the feasibility of using orchid protoplasts for protein–protein interaction studies, the construct containing the *PaCDKA* gene fused N-terminal half of the *EYFP* (*N*-*(nEYFP)*-*CDKA1*) was co-transfected with a construct containing *PaCYCD3;1* fused to a C-terminal half of the *EYFP* (*N*-*cEYFP*-*CYCA3;1*). The direct interaction of PaCDKA and PaCYCD3;1 scored by the presence of yellow fluorescence was verified by confocal microscopy (**Figure 3A** and Supplementary Figure S3A). As a negative control, the construct containing *N*-*(nEYFP)*-*CDKA1* was co-transfected with only the C-terminal half of the EYFP (*N*-*cEYFP*) construct. No fluorescence was detected when the *N*-*cEYFP* construct was co-transfected with the *N*-*(nEYFP)*-*CDKA1* construct (**Figure 3B** and Supplementary Figure S3B). This is consistent with biolistic-based BIFC assay in petal cells (Lin H.Y. et al., 2014).

### PaE2F3/PaDP2 Activates Expression of S- and G2-Phase Cell Cycle Genes

The cell division cycle is fundamental for the growth of organisms (Hall et al., 2004). In plants, cell cycle genes are duplicated and diverged to accommodate complex developmental programs (Gutierrez, 2016). Our previous study showed that expression of the core cell cycle genes is coordinately regulated from ovule development to embryogenesis during sexual reproduction in *P*. *aphrodite* (Lin H.Y. et al., 2014). Moreover, transcripts associated with cell cycle-associated biological processes, such as DNA replication initiation, cell division, and regulation of the cell cycle are strongly enriched in interior ovary tissues 30–40 days after pollination (DAP) when ovules are developing (Fang et al., 2016). Among the enriched cell cycle regulators identified, *PaE2F3* encodes an evolutionarily conserved transcription factor that heterodimerizes with the DP protein to control G1/S transition as cells enter the division cycle. Being the cell cycle activator, E2F/DP transcription factor is reported to activate genes involved in DNA replication and mitotic functions (Ishida et al., 2001; Ramirez-Parra et al., 2003; Fung and Poon, 2005; Vandepoele et al., 2005; Dante et al., 2014). Furthermore, overexpression of *Arabidopsis* E2F (AtE2Fb) and its dimerization partner AtDPa is sufficient to activate downstream cell cycle genes and drive cell proliferation in differentiated tissues (De Veylder et al., 2002; Rossignol et al., 2002; Magyar et al., 2005; Sozzani et al., 2006). To investigate the potential targets of PaE2F3, the expression levels of selected cell cycle regulators enriched simultaneously in ovary tissues at 30–40 DAP (**Table 3**) were analyzed in protoplast cells co-transfected with *PaE2F3* and its interaction partner *PaDP1* or *PaDP2* (Lin H.Y. et al., 2014).

**Table 3:** Comparative transcript abundances of cell cycle genes in reproductive tissues of *P. aphrodite* by RNA-sequencing analysis.

		FPKM values										
		30/40	50/60	70/80	90/100/120	140/160	180/200	PLB	Protocorm	Young	Stalk	Floral
Transcript ID	Annotation	DAP	DAP	DAP	DAP	DAP	DAP			leaves	buds	stalks
**E2Fs and DPs**									
orchid.id113590.tr318945	PaE2F1	6.7	4.3	2.9	7.8	2.2	1.5	5.0	7.0	3.8	3.0	3.7
orchid.id117614.tr38827	PaE2F2	3.1	1.4	1.5	1.5	1.3	2.0	0.3	2.5	1.1	0.7	0.9
orchid.id1949.tr77229	PaE2F3	14.2	6.4	2.7	1.8	5.5	4.8	3.2	5.2	3.4	3.4	4.6
orchid.id42993.tr191185	PaE2F4	1.3	0.9	1.3	0.9	0.8	0.5	1.7	2.6	2.8	2.4	2.9
orchid.id113906.tr107393	PaDP1	53.0	48.1	54.5	60.2	32.4	37.7	47.5	41.7	38.6	43.1	42.6
orchid.id123685.tr127191	PaDP2	4.4	4.5	3.1	3.4	3.0	3.0	1.2	1.4	3.1	1.9	2.8
**Cell cycle genes enriched at 30/40 DAP**									
orchid.id130751.tr142269	PaPCNA1	44.1	27.2	4.9	3.9	11.1	5.7	38.1	22.2	18.0	15.4	28.7
orchid.id93462.tr632091	PaCYCA1;1	22.2	16.3	0.4	2.0	6.9	4.5	5.3	25.9	17.7	0.2	7.8
orchid.id104694.tr178804	PaCYCA2;3	20.3	12.0	1.8	1.8	7.6	15.1	7.5	9.2	9.2	6.0	8.5
orchid.id119353.tr176450	PaCYCA3:2	11.3	6.3	1.4	0.2	0.4	0.1	2.8	5.1	4.3	2.6	7.0
orchid.id130531.tr130848	PaCYCB1;1	27.5	22.3	1.1	2.4	1.8	0.1	1.7	3.2	11.5	14.9	31.9
orchid.id3686.tr138153	PaCYCB1;2	4.2	5.7	0.2	0.2	0.0	0.0	0.1	0.2	3.2	0.0	2.1
orchid.id100343.tr56122	PaCYCB2;1	34.8	26.1	0.2	1.2	0.8	0.0	2.8	4.2	8.9	0.3	12.5
orchid.id121744.tr208045	PaCYCD1;3	7.7	6.0	1.9	0.6	0.6	0.6	0.6	0.3	5.2	0.2	2.8

**FIGURE 4:**
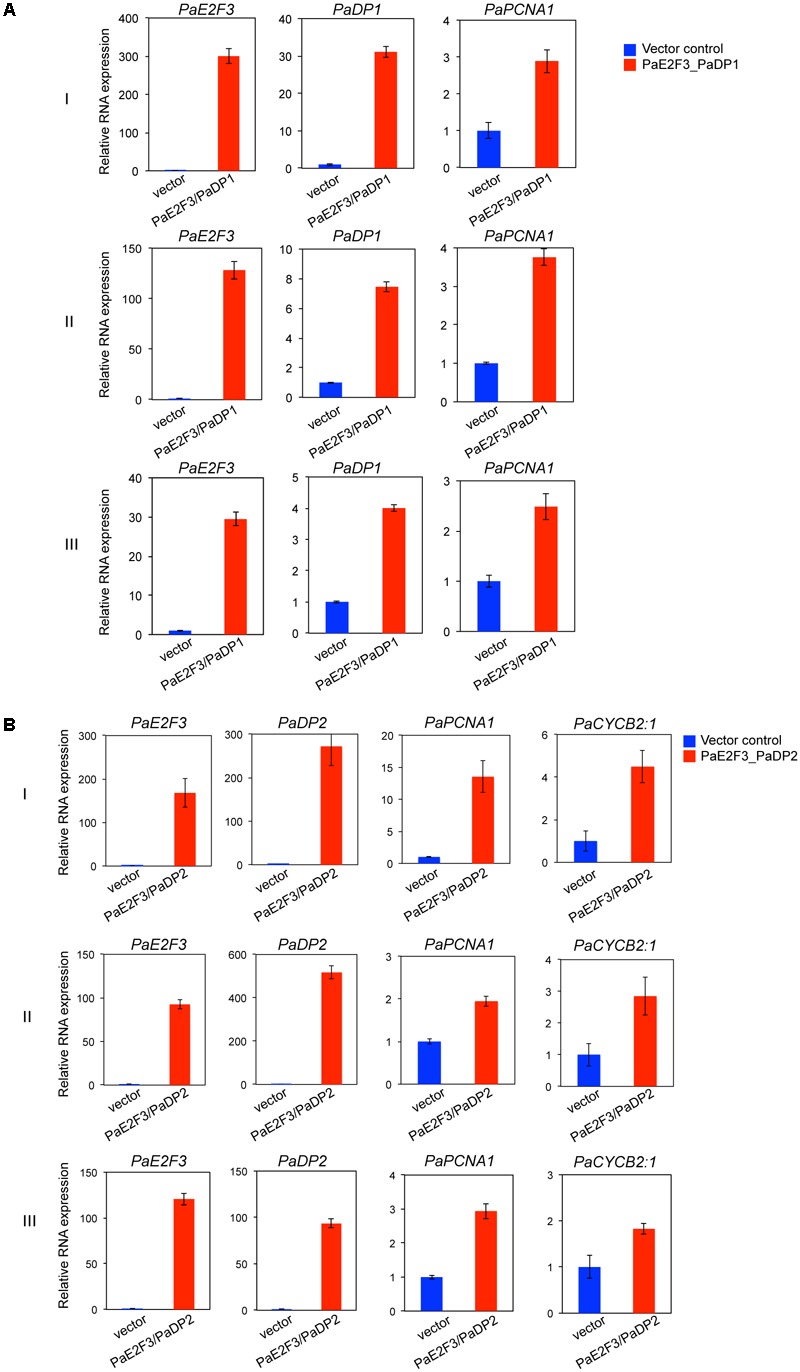
Overexpression of PaE2F3 and PaDP2 up-regulates expression of the specific cell cycle genes. **(A)** Validation of expression of *PaE2F3* and *PaDP2* mRNAs in protoplast cells co-transfected with the *PaE2F3* and *PaDP2* constructs. **(B)** Relative expression levels of *PaPCNA1* and *PaCYCB2:1* in protoplast cells co-transfected with the *PaE2F3* and *PaDP2* constructs. Protoplasts transfected with the empty vectors were used as a negative control. Expression levels of the indicated genes from protoplasts transfected with the empty vectors (vector) were arbitrarily set to be one. Three independent experiments I, II, and III are shown.

After transfection, overexpression of *PaE2F3* and *PaDP1* was verified by quantitative RT-PCR analysis (**Figure 4A**). Overexpression of *PaE2F3* and *PaDP1* resulted in an increase in expression levels of *PaPCNA1* in three independent experiments (**Figure 4A**), but did not have significant effects on expression of *PaCYCB2;1, PaCYCA1:1, PaCYCA2:3, PaCYCA3:2, PaCYCB1:1*, and *PaCYCD1:3* (Supplementary Figure S4A). Because *PaCYCB1;2* mRNA could not be reliably detected in transfected protoplast cells, it was omitted from further analysis.

For protoplast cells transfected with *PaE2F3* and *PaDP2* constructs, accumulation of *PaE2F3* and *PaDP2* mRNAs was also verified by quantitative RT-PCR analysis (**Figure 4B**). Overexpression of *PaE2F3* and *PaDP2* up-regulated expression of *PaPCNA1* and *PaCYCB2;1* (**Figure 4B**) but did not have significant effects on accumulation of *PaCYCA1:1, PaCYCA2:3, PaCYCA3:2, PaCYCB1:1*, and *PaCYCD1:3* (Supplementary Figure S4B). Activation of *PaPCNA1* and *PaCYCB2;1* by co-overexpression of *PaE2F3* and *PaDP2* was validated in three independent experiments (**Figure 4B**). Hence, our data provide evidence that *PaPCNA1* and *PaCYCB2;1* are the potential targets of PaE2F3.

### The *DR5v2* Reporter Is Responsive to Auxin in *Phalaenopsis* Protoplasts

**FIGURE 5:**
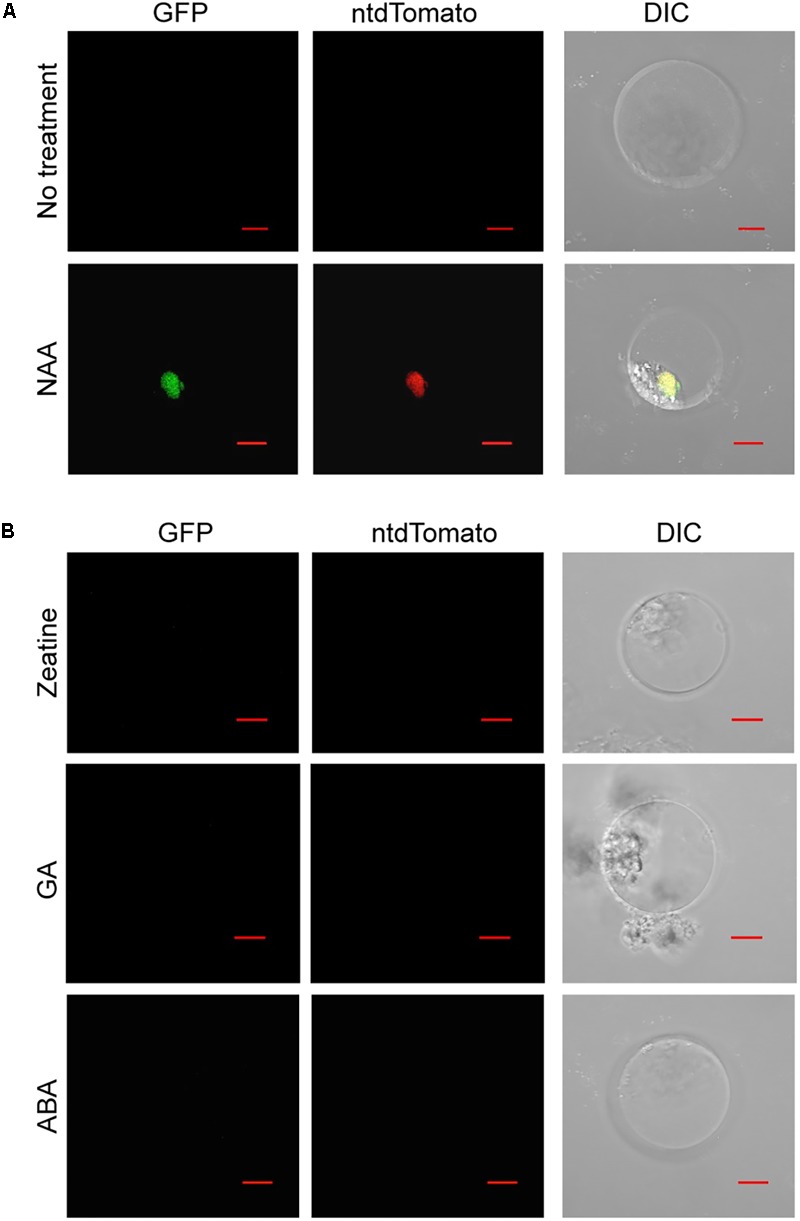
Auxin activates the *DR5v2* reporter in *Phalaenopsis* protoplast transient expression assay. **(A)** NAA activated *DR5v2* reporter. **(B)** Zeatin, GA, and ABA did not activate *DR5v2* reporter. DIC, differential interference contrast images of cells superimposed with GFP and ntdTomato signals. Scale bar, 10 μm.

To assess the potential of using *Phalaenopsis* protoplasts to investigate the hormone response, the auxin reporter *DR5v2* (Liao et al., 2015) was transfected into the petal protoplasts. While treatment with a synthetic auxin, NAA, resulted in accumulation of GFP and ntdTomato florescent proteins in the nuclei, only the background signal was detected in the absence of NAA treatment (**Figure 5A** and Supplementary Figure S5). Furthermore, treatment with trans-zeatin, GA, or ABA had no effect on accumulation of GFP and ntdTomato florescent proteins in the transfected protoplasts (**Figure 5B** and Supplementary Figure S5). Taken together, these results demonstrated that *DR5v2* reporter responds to the auxin signal in petal protoplasts and suggests the potential of using *DR5v2* reporter to map the auxin regulatory pathway in *Phalaenopsis* orchids.

## Discussion

Protoplast transient expression systems are a powerful tool for studying the molecular mechanisms underlying signal transduction pathways. Here, we described a streamlined protocol for petal protoplast isolation and polyethylene glycol-calcium transfection for *P. aphrodite* (**Figure 1A**) and demonstrated its feasibility for analyses of protein subcellular localization and protein–protein interaction. Moreover, our reported transfection efficiency (> 80%) is significantly improved in comparison to the transfection efficiency of *Phalaenopsis* protoplasts reported recently (Li et al., 2018). The ability to isolate large numbers of viable protoplasts (**Table 1**), high transfection efficiency, and high numbers of viable transfected cells (**Table 2**) enable construction of a hierarchical gene regulatory pathway and studies of hormone responses. Hence, this protocol provides a useful platform for in-depth studies of gene functions and molecular networks that were previously difficult in *Phalaenopsis* orchids. Recently, protoplast transient expression systems have been extended to studies on dissection of miRNA pathways (Martinho et al., 2015), protein-DNA binding (Lee et al., 2017), microbe-associated molecular patterns-triggered immunity (Fraiture et al., 2014; Kanofsky et al., 2016), ribonuclease-mediated mRNA decay (Hayashi et al., 2016), and auxin-mediated transcriptional regulatory networks (Wehner et al., 2017). It is therefore conceivable that our established protocol may be applied to diverse aspects of orchid biology studies.

### Co-expression of *PaE2F3*/*PaDP1* and *PaE2F3*/*PaDP2* Induce Expression of *PaPCNA1*

PaE2F3 has been shown to interact with PaDP1 and PaDP2 in yeast two-hybrid and petal transient expression assays (Lin H.Y. et al., 2014). As proof-of-principle for gene regulation study, we found that transient overexpression of *PaE2F3*/*PaDP2* transcription factor was capable of up-regulating expression of *PaPCNA1* and *PaCYCB2;1* that are co-expressed with *PaE2F3* in ovary tissues of *P. aphrodite*. Transient overexpression of *PaE2F3*/*PaDP1* transcription factor, on the other hand, only up-regulated expression of *PaPCNA1* and did not seem to have a significant effect on the expression of the other genes tested. It is possible that PaE2F3 interacts with different partners to regulate both overlapping and distinct sets of cell cycle genes in *P. aphrodite*.

It is not surprising to see that co-expression of *PaE2F3* and *PaDPs* induced accumulation of *PaPCNA1* mRNA because PCNA, which is required for DNA replication in both plant and animal systems, is identified as the direct downstream target of E2F/DP protein (Yamaguchi et al., 1995; Egelkrout et al., 2002; Kosugi and Ohashi, 2002; Vandepoele et al., 2005). It is intriguing to discover that PaE2F3/PaDP2 transcription factor induced accumulation of B-type cyclin, *PaCYCB2;1*, which is normally considered to be an M phase gene (Van Leene et al., 2011). The functional roles of E2F/DP at the G2-M checkpoint and mitotic activity have been reported in animals (Ren et al., 2002; Li et al., 2012). In *Arabidopsis*, E2Fa-DPa transcription factor has been reported to regulate G2-M phase gene *CDKB1;1* to control stomatal development (Boudolf et al., 2004). Our findings illustrate a potential PaE2F3/PaDP2-mediated cross talk between the G1-S and G2-M stages in ovary development of *P. aphrodite* and support the role of E2F in G2-M phase. It will be interesting to investigate how PaE2F3/PaDP2 regulates expression of *PaCYCB2;1* to coordinate the cell cycle progression in *Phalaenopsis* orchids. Taken together, our data demonstrate the possibility of using the orchid protoplast transient expression system to quickly screen for potential E2F/DP downstream targets in developing ovary tissues.

### The Auxin Reporter *DR5v2* Is Functional in *Phalaenopsis* Orchids

Auxin is reported to play important roles in orchid development (Arditti et al., 1971; Tsavkelova et al., 2007; Novak and Whitehouse, 2013; Novak et al., 2014). However, it is not clear how auxin responses are regulated to coordinate specialized orchid developmental programs. *DR5v2* reporter assay provides evidence that petal protoplasts may be potentially conducive to dissecting the auxin response pathway of *Phalaenopsis* orchids. In addition, the orchid protoplast transient expression system could be used to assess the transcriptional responses of auxin signaling molecules or regulatory components.

### Orchid Protoplasts: One for All?

In addition to *Phalaenopsis* orchids, petal protoplasts have successfully been used for gene functional studies in petunia and rose (Faraco et al., 2011; Hirata et al., 2012) supporting the general applicability of the petal system. However, conceivable limitations of the petal protoplast transient expression system may exist. It is reported that protoplasts retain their differentiation state and gene expression program within the time frame of transient expression experiments (Faraco et al., 2011; Lin Y.C. et al., 2014). Accordingly, protoplasts with the right cellular context providing the ideal gene expression program may be required to address tissue- or context-specific questions. It is possible that petal protoplasts are not suitable to analyze light- and sugar-dependent responses of the photosynthetic genes. In such cases, results should be interpreted with caution. It is presently difficult to isolate active protoplasts from mature leaf tissues, the most used source for protoplast isolation. During the attempts to isolate mesophyll protoplasts, protoplasts collapsed hours after isolation regardless the mannitol concentration tested. It is not clear why leaf mesophyll protoplasts are sensitive to the isolation procedure. Generally, protoplasts rupture in hypotonic solution and collapse in hypertonic solution (Ohshima and Toyama, 1989). It is possible that the cell wall of mesophyll cells releases phytotoxic factors that poison the isolated protoplasts (Hahne and Lorz, 1988). The presence of abundant calcium oxalate crystals may also contribute to the damage to protoplasts. The establishment of orchid protoplast transient expression protocols for different tissue types in the future may be important to address tissue- or context-specific questions.

Protoplast-based high-throughput systems have been established for dissection of signaling pathways, analysis of transcription factor functions, and identification of kinase-associated phosphoisoforms (Wehner et al., 2011; Zhang et al., 2015; Dory et al., 2016; Wehner et al., 2017). Therefore, the orchid protoplast transient expression system provides a molecular tool to characterize functions of proteins identified from open reading frames from recently released transcriptome datasets (Cai et al., 2015; Fang et al., 2016; Chao et al., 2017) and potentially allows discovery and/or validation of hierarchical gene regulatory networks. In summary, the protocol presented here will enable in-depth studies of the molecular networks governing the unique developmental processes and hormone regulating pathways in orchids and provide an alternative method to conduct functional genomic studies in *Phalaenopsis* orchids.

## Author Contributions

S-CF wrote the manuscript and designed the experiments. H-YL developed the protoplast isolation and transfection system. J-CC maintained the orchid plants and did the RT-PCR experiments. All authors read and approved the final manuscript.

## Conflict of Interest Statement

The authors declare that the research was conducted in the absence of any commercial or financial relationships that could be construed as a potential conflict of interest.
