# Supplementary material for: A Protoplast Transient Expression System to Enable Molecular, Cellular, and Functional Studies in Phalaenopsis orchids
Source: Front Plant Sci. 2018 Jun 22;9:843. doi: 10.3389/fpls.2018.00843 (PMC6024019; doi:10.3389/fpls.2018.00843)
Supplement: Supplementary file 1 [file Table_1.DOCX]

**Supplementary Table S1. Primer pairs used for qPCR**

| **Gene name** | **Forward primer** | **Reverse primer** | **Amplicon size (bp)** | **Annealing temp. (**°C) |
| --- | --- | --- | --- | --- |
| *PaE2F3* | 5′-GATTTTATGGGTGGCAGTGG-3′ | 5′-CATTGCTCAGATGGTGTGCT-3′ | 128 | 58 |
| *PaDP1* | 5′-AGGCAGAACGTGCTTCTCTC-3′ | 5′- GGTAGAGCCACTCCACCTGA-3′ | 154 | 58 |
| *PaDP2* | 5′-GGCCACCGTTGAAATAGAGA-3′ | 5′-AGCAGCTGATAGTGGGCATC-3′ | 127 | 58 |
| *PaPCNA1* | 5′-GGGATATTGGAACTGCGAAC-3′ | 5′-AACAGCCTCGGAAAGTGAAG-3′ | 158 | 58 |
| *PaCYCA1;1* | 5′-GCCGAACTATCACTCCCAGA-3′ | 5′-ATGCGTAAGAGTTGCGTTCC-3′ | 129 | 58 |
| *PaCYCA2;3* | 5′-ATGGGACCATGCAAAACAATA-3′ | 5′-GTAGAGGCTGCACATTTGAGG-3′ | 140 | 58 |
| *PaCYCA3;2* | 5′-CATGCTCATTGCCTCAAAGT-3′ | 5′-GCCTTCAAGAACCTCCTCAG-3′ | 183 | 58 |
| *PaCYCB1;1* | 5′-TCGTAGCAAGGTTGCTTGTG-3′ | 5′-ATGGCTCTCATCTTCGCATT-3′ | 177 | 58 |
| *PaCYCB2;1* | 5′-TGGTGGATTTTCACAAGCAG-3′ | 5’-CGGACAGCATTGAATCAAGA-3′ | 125 | 58 |
| *PaCYCD1;3* | 5′-GCCAGCTGATGGACCAGTAT-3′ | 5′-GGTGAAGGACTTCTGGGTGT-3′ | 140 | 58 |
| *PaUBI1* | 5′-AACTCCATCGCCTTCCTCTT-3′ | 5′-TGAAGCATGGCATCAATTTC-3′ | 101 | 58 |
