# Supplementary figures and images for: A Protoplast Transient Expression System to Enable Molecular, Cellular, and Functional Studies in Phalaenopsis orchids
Source: Front Plant Sci. 2018 Jun 22;9:843. doi: 10.3389/fpls.2018.00843 (PMC6024019; doi:10.3389/fpls.2018.00843)

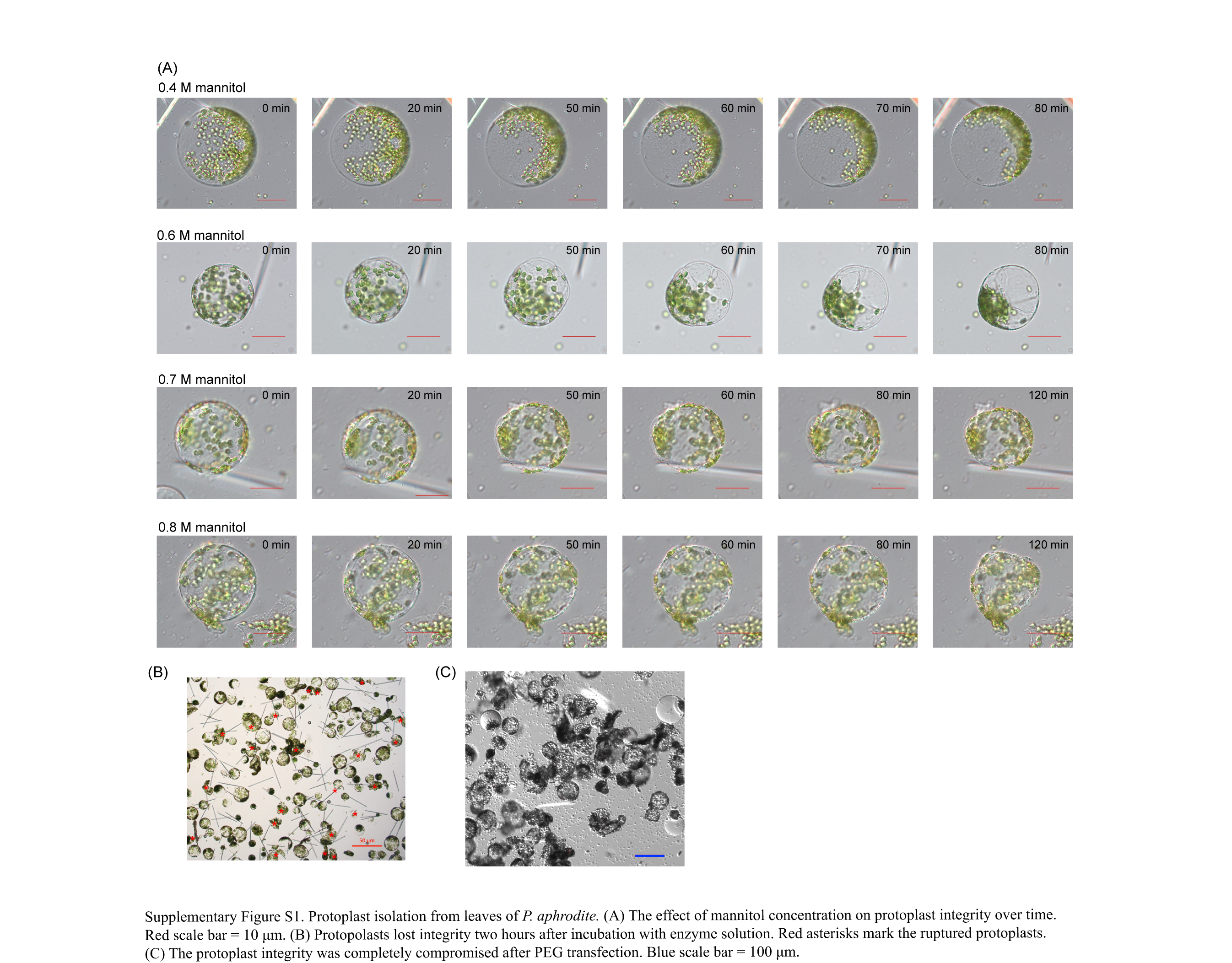

Supplement: Supplementary file 4 [file Image_1.TIF]

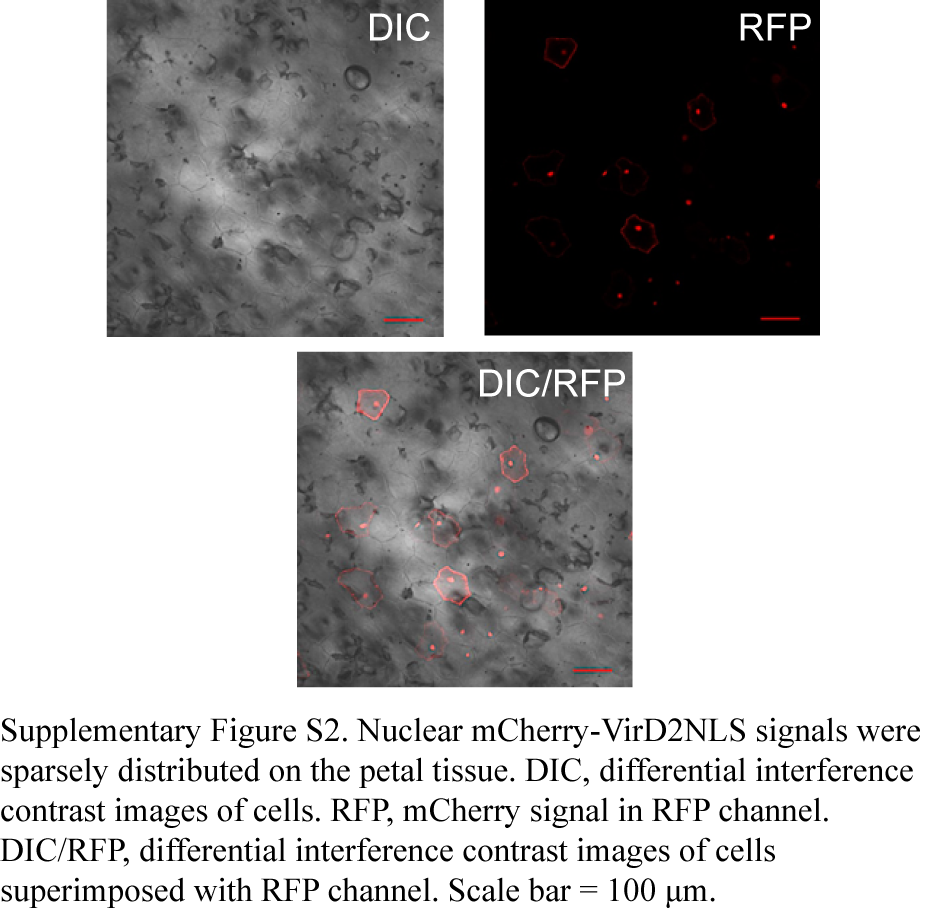

Supplement: Supplementary file 5 [file Image_2.TIF]

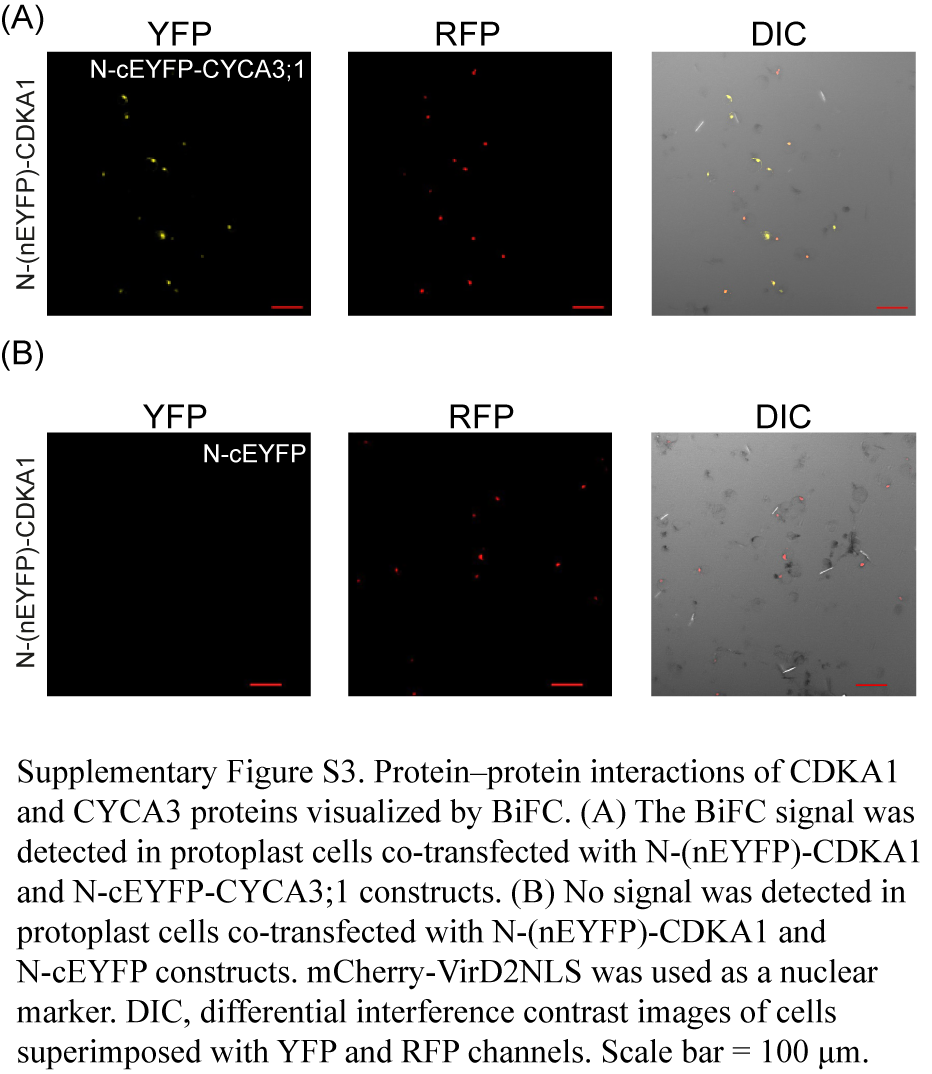

Supplement: Supplementary file 6 [file Image_3.TIF]

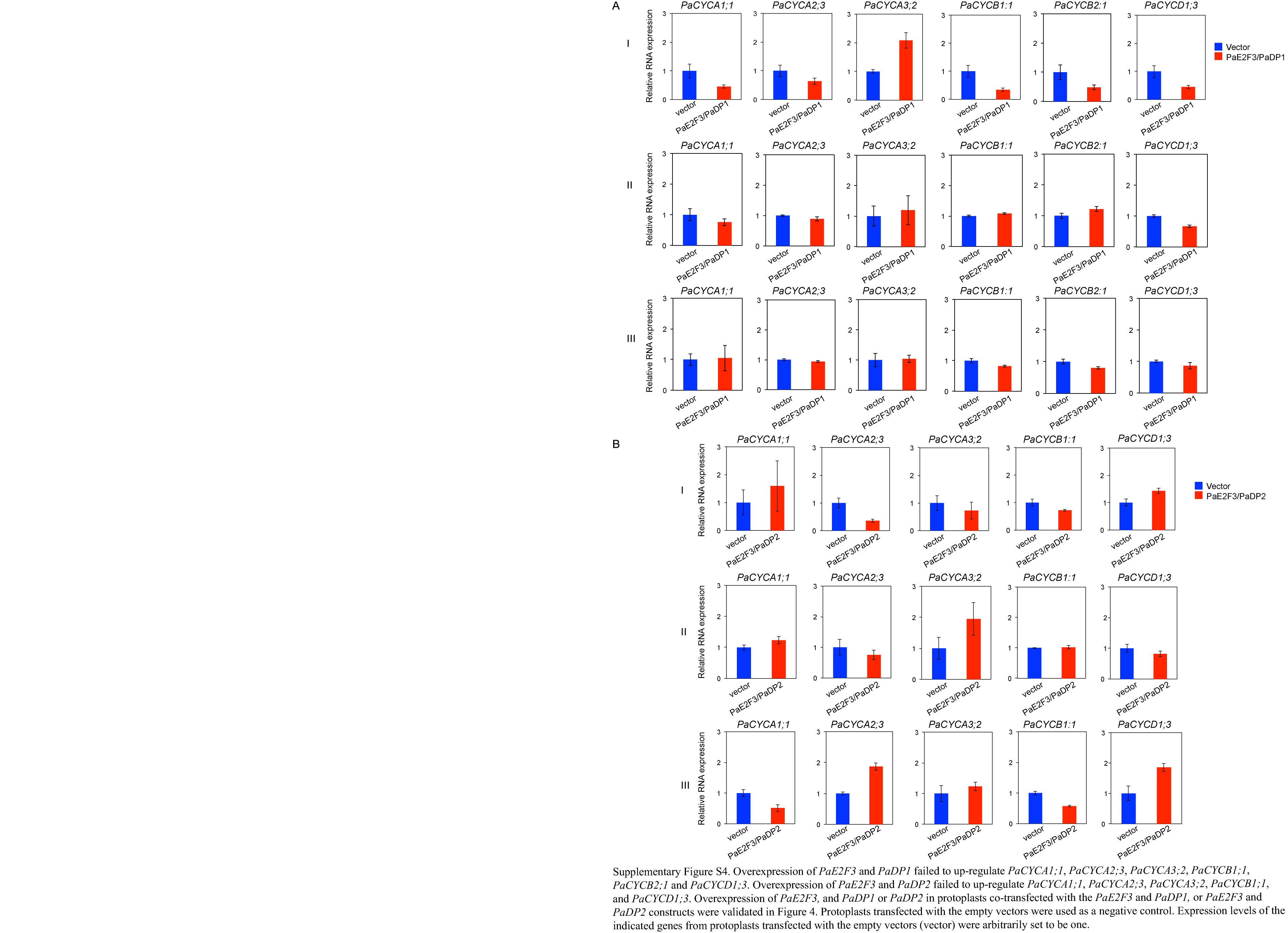

Supplement: Supplementary file 7 [file Image_4.TIF]

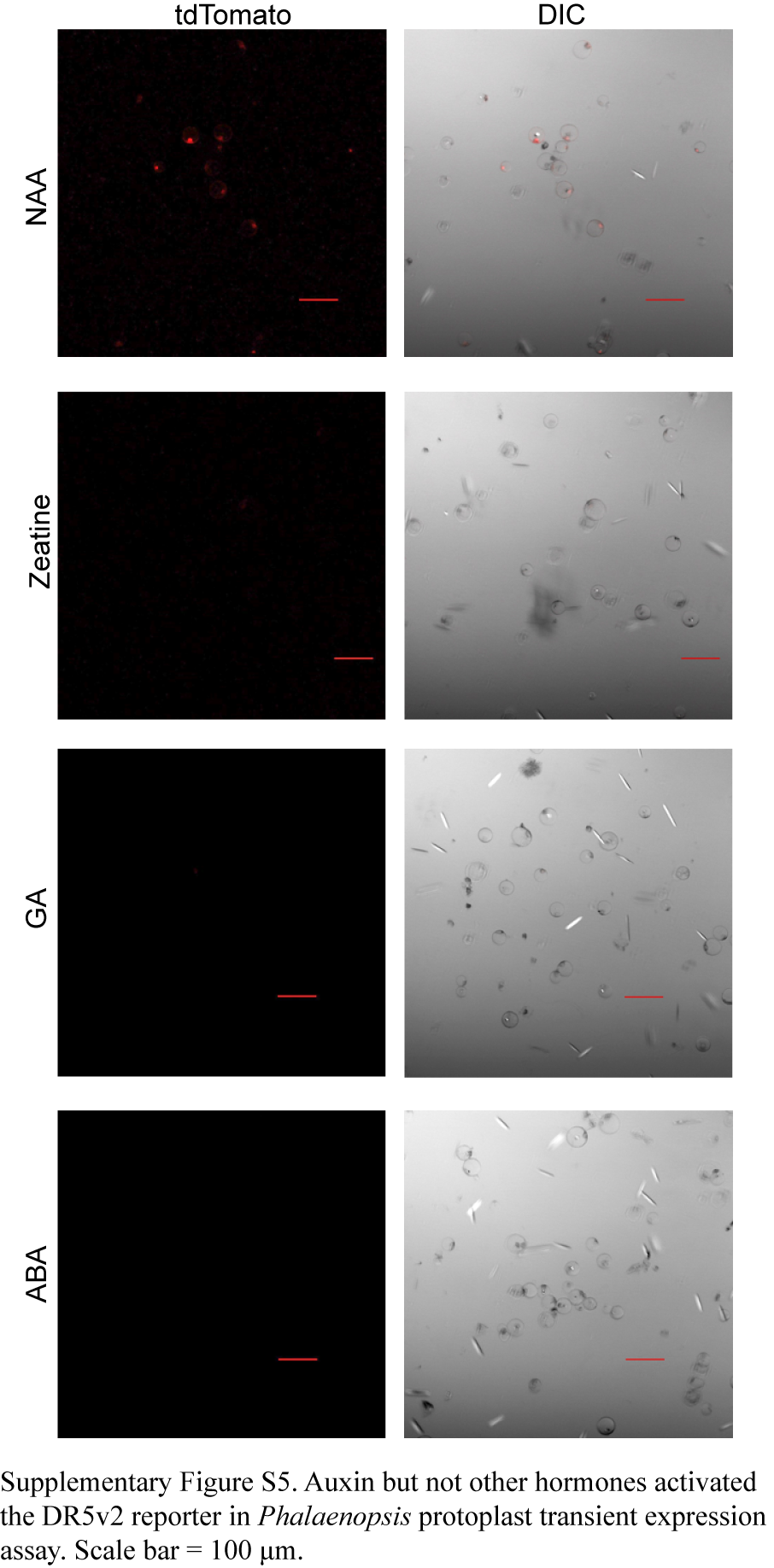

Supplement: Supplementary file 8 [file Image_5.TIF]
